# Silk fibroin, gelatin, and human placenta extracellular matrix-based composite hydrogels for 3D bioprinting and soft tissue engineering

**DOI:** 10.1186/s40824-023-00431-5

**Published:** 2023-11-17

**Authors:** Karl Heinrich Schneider, Benjamin J. Goldberg, Onur Hasturk, Xuan Mu, Marvin Dötzlhofer, Gabriela Eder, Sophia Theodossiou, Luis Pichelkastner, Peter Riess, Sabrina Rohringer, Herbert Kiss, Andreas H. Teuschl-Woller, Vincent Fitzpatrick, Marjan Enayati, Bruno K. Podesser, Helga Bergmeister, David L. Kaplan

**Affiliations:** 1 Center for Biomedical Research and Translational Surgery, Medical University of Vienna, 1090 Vienna, Austria; 2 Ludwig Boltzmann Institute for Cardiovascular Research, Vienna, 1090 Austria; 3 Department of Biomedical Engineering, Tufts University, Medford, MA 02155 USA; 4 Roy J Carver Department of Biomedical Engineering, College of Engineering, the University of Iowa, Iowa City, IA 52242 USA; 5 Department of Mechanical and Biomedical Engineering, Boise State University, Boise, ID 83725 USA; 6 Department of Obstetrics and Gynecology, Division of Obstetrics and Feto-Maternal Medicine, Medical University of Vienna, 1090 Vienna, Austria; 7 Department Life Science Technologies, University of Applied Sciences Technikum Wien, 1200 Vienna, Austria; 8 UMR CNRS 7338 Biomechanics & Bioengineering, Université de Technologie de Compiègne, Sorbonne Universités, 60203 Compiegne, France

## Abstract

**Background:**

There is a great clinical need and it remains a challenge to develop artificial soft tissue constructs that can mimic the biomechanical properties and bioactivity of natural tissue. This is partly due to the lack of suitable biomaterials. Hydrogels made from human placenta offer high bioactivity and represent a potential solution to create animal-free 3D bioprinting systems that are both sustainable and acceptable, as placenta is widely considered medical waste. A combination with silk and gelatin polymers can bridge the biomechanical limitations of human placenta chorion extracellular matrix hydrogels (hpcECM) while maintaining their excellent bioactivity.

**Method:**

In this study, silk fibroin (SF) and tyramine-substituted gelatin (G-TA) were enzymatically crosslinked with human placental extracellular matrix (hpcECM) to produce silk-gelatin-ECM composite hydrogels (SGE) with tunable mechanical properties, preserved elasticity, and bioactive functions. The SGE composite hydrogels were characterized in terms of gelation kinetics, protein folding, and bioactivity. The cyto- and biocompatibility of the SGE composite was determined by in vitro cell culture and subcutaneous implantation in a rat model, respectively. The most cell-supportive SGE formulation was then used for 3-dimensional (3D) bioprinting that induced chemical crosslinking during extrusion.

**Conclusion:**

Addition of G-TA improved the mechanical properties of the SGE composite hydrogels and inhibited crystallization and subsequent stiffening of SF for up to one month. SGE hydrogels exhibit improved and tunable biomechanical properties and high bioactivity for encapsulated cells. In addition, its use as a bioink for 3D bioprinting with free reversible embedding of suspended hydrogels (FRESH) has been validated, opening the possibility to fabricate highly complex scaffolds for artificial soft tissue constructs with natural biomechanics in future.

**Graphical Abstract:**

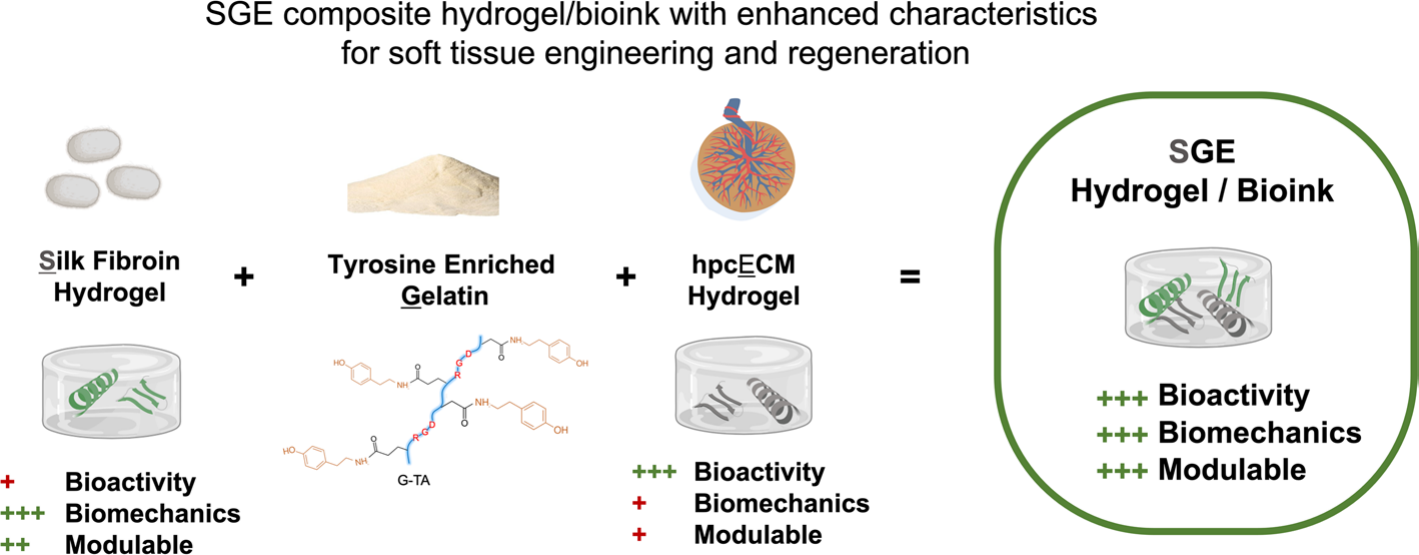

**Supplementary Information:**

The online version contains supplementary material available at 10.1186/s40824-023-00431-5.

## Introduction

Soft tissue engineering presents inherent challenges and limitations in translational applications. A core challenge is providing and maintaining mechanically stable and soft artificial tissue constructs ex vivo with high fidelity to the defective target tissue in vivo. These tissue constructs support soft tissue regeneration by providing a microenvironment that enables cell migration, proliferation and, ultimately, adequate extracellular matrix (ECM) production within the scaffold [1–3]. To meet these criteria and to prevent scaffold collapse, tuning biomaterial and compositional features is needed. Current biopolymer formulations such as gelatin/collagen or alginate do not meet the requirements to serve as complex constructs for cell-based tissue engineering. Compromises with these biopolymer options can include reduced biomechanics, low bioactivity, or poor architectural complexity. To address these limitations, in the present study, we developed novel compositions of silk fibroin (SF), chemically modified gelatin and placental ECM (SGE) hybrid hydrogels to address the design criteria for 3D bioprintable biomaterial/bioinks for soft tissue engineering.

An optimal bioink should have similar biological, rheological and mechanical characteristics to the target soft tissues. The rheological behavior of the bioink is a critical property, as this impacts cell proliferation, differentiation, or apoptosis [4]. In this respect, viscosity and shear-thinning characteristics must be adjusted to support the 3D printing extrusion process and allow structural stability of the printed constructs, without negatively influencing cell viability. Additionally, gelation time should be regulated to form self-supportive constructs, while the matrix itself must provide a biological environment that encourages cell viability and exchange between metabolic byproducts (nutrients and metabolic byproducts). Porous 3D hydrogel networks with high water content offer optimal properties to mimic soft tissue constructs for tissue engineering and regeneration [2]. The physical, chemical and biological properties of these hydrogels can be tailored to match design goals.

Hydrogel mechanical properties are dependent on the type and concentration of polymer utilized [5, 6] and can be classified as synthetic or natural based on the nature of polymers [7]. Synthetic polymers such as poly(2-hydroxyethyl methacrylate) (PHEMA), polyethylene glycol (PEG) or polylactic acid (PLA) possess good mechanical strength, but lack bioactivity and in some cases biocompatibility due to inflammatory responses in vivo. Hydrogels consisting of natural polymers, such as collagen [8], hyaluronic acid [9], dextran [10], chitosan [11], or decellularized tissue (ECM) [12, 13], are desirable due to their biocompatibility and biodegradability, but often lack sufficient strength to function directly as tissue substitutes.

Silk is a promising natural protein biomaterial with outstanding mechanical characteristics and biocompatibility, making it a great choice for developing extrudable bioinks for 3D bioprinting [14–18]. As an example, SF harvested from cocoons of the domesticated silkworm *Bombyx mori*, possess the aforementioned properties in addition to having controllable porosity and low immunogenicity in sponge and hydrogel forms [19]. However, unlike ECM- or collagen-based hydrogels, SF hydrogels have two inherent characteristics which can limit its use as bioscaffolds. First, SF hydrogels show low bioactivity as they possess only a small number of cell-binding sequences [20]. As a result, cell–matrix interactions are impaired by the lack of integrin-binding motifs, preventing cell adherence and spreading within the SF hydrogels. Second, SF hydrogels tend to stiffen over time due to the formation of beta (β)-sheet structures (crystallization) [21]. β-sheets are responsible for the biomechanical strength of SF scaffolds as sponges [22], thin films [23] or fibers [24]. However, β-sheets also result in loss of elasticity, causing the SF hydrogels to embrittle over time. Therefore, one aim of this study was to prevent crystallization over time and to maintain the elastic mechanical properties as a key to generate functional soft tissue constructs in future. Various methods have been developed to achieve this goal before [25]. Here, we used chemical crosslinking which usually leads to slow gelation times of SF hydrogels when using cell-friendly concentrations of enzymatic crosslinking agents, such as < 10 IU/mL horseradish peroxidase (HRP) and < 0.01% hydrogen peroxide (H_2_O_2_). This translates to longer required incubation times (up to several hours) before the cell laden 3D-printed constructs can be immersed by culture medium, resulting in stress to the encapsulated cells and reducing cell functions. Therefore, tuning hydrogels to increase rates of gelation to achieve their final strength within a short time after printing (i.e. < 1 h) is important.

A strategy that overcomes all these challenges in developing a mechanically stable and bioactive SF hydrogel with rapid gelation times is generating a composite SF-based hydrogel, where an additional material is combined with silk to retain the desirable properties of the SF while enhancing elasticity and rates of gel stabilization [26].

In this study, we developed a silk-based composite bioink for 3D bioprinting to form into hydrogels. This gel harnesses the superior biomechanical properties of SF alongside enhanced cell interactions inside the matrix, facilitated by the inclusion of human chorionic ECM. Printability of such a soft but mechanically stable bioink can provide new soft tissue structures. We have previously demonstrated the modulation of SF hydrogel gelation by mixing with tyramine-enriched gelatin (G-TA) [27]. We observed that adding binding sites in a SF hydrogel can delay crystallization and preserve the elastic behavior of the hydrogels, however, hydrogel bioactivity outcomes could be improved. In the present study, SF and G-TA gels were crosslinked enzymatically with a human placental chorionic ECM (hpcECM) gel [28] to improve cell–matrix interactions by introducing additional cell adhesion motifs. To explore the effect of each constituent on the material properties, three different blends of silk/ECM/G-TA ratios/formulations were developed. Chemical, mechanical and cell–matrix interactions were evaluated to compare these SGE blends with each other.

The viscosity of the SGE solutions before gelation was low and therefore less suitable for classical extrusion-based 3D bioprinting where spatial integrity throughout the extrusion process is required. Recently, an innovative extrusion-based bioprinting technique was developed that is capable of printing soft, cell-encapsulating and low viscosity bioinks, based on a freeform reversible embedding of suspended hydrogels or FRESH [29]. Here the extruded material is continuously surrounded by the support structure [30]. Printing in 3 directions (x, y, z) allows for control over shape and cellular distribution within the bioink. Combined with the optimized mechanical and biological properties of SF, FRESH printing of SF-containing bioinks offers new directions for the 3D printing process for soft-tissues.

In this study, the original FRESH bioprinting method was further optimized to work with our SGE material. Specifically, biomaterial scaffolds were extruded into a supporting hydrogel bath to keep the bioink in place until gelation was complete. The two crosslinking components (HRP and H_2_O_2_) react upon mixing in the presence of the SGE bioinks. To prevent printability challenges and preserve the shape fidelity of the final constructs, the crosslinkers were spatially separated by confining one to the bioink (HRP) and the other to the support bath (H_2_O_2_). Following printing, the support hydrogel can be extracted due to its thermosensitivity at 37 °C, resulting in self-standing SGE 3D constructs. We want to show that with the SGE hydrogels the advantages of the individual components can be mutually complemented. With FRESH bioprinting technology and SGE bioinks, we will be able to print complex shapes to create tissue scaffolds with high biocompatibility and potential for special biomechanical requirements to mimic native tissue.

## Material and methods

### Synthesis of hpcECM, SF, and G-TA materials

Placentas were harvested after planned caesarian section deliveries at term (pregnancy week 37 + 0 to 40 + 0). All donors were serologically tested for HIV, HBV and HCV. Human placental chorion extracellular matrix hydrogels were fabricated as described previously [28]. Briefly, chorionic tissue was separated from the other tissue parts and frozen to -80 °C for at least 24 h prior use. Pooled chorion material was chopped into small pieces and decellularized by intense washing steps using 2% Triton X-100 and 0.02% ethylenediaminetetraacetic acid for 3 subsequent days, with a daily intermediate PBS washing step to remove debris and blood products, as described previously [28]. DNA residuals were removed by an overnight 0.04% DNAse (Sigma-Aldrich, Austria) treatment. Following an additional washing step with PBS, the decellularized tissue was sterilized with 0.18% paracetic acid in 4.8% ethanol for 2.5 h, frozen at -20 °C, lyophilized and cryomilled with a FreezerMill 6770 (SpexSamplePrep, USA). Specimens from decellularized chorionic tissue were examined by histological staining and biochemical quantification methods to confirm cell removal and preservation of ECM components post-decellularization as described previously [31, 32]. Briefly, DNA was extracted using a Tissue DNA Extraction Mini Kit (FavorprepTM, Taiwan) and quantified using an AccuBlue® High Sensitivity dsDNA Quantitation Kit (Biotium, USA). Collagen content in the pre-gel samples was determined by measuring the hydroxyproline content of neutralized samples after hydrolysis in 6 M HCl at 95 °C for 20 h. For direct measurement of sulfated glycosaminoglycan (GAG) content, dilutions of the pre-gel samples were stained with 1,9-dimethyl methylene blue (DMB, Sigma-Aldrich, Austria) and measured photometrically at 525 nm. A fastin-elastin assay (Biocolor, UK) was performed to examine elastin levels according to the manufacturer's protocol.

The chorion ECM powder was solubilized by enzymatic digestion under acidic conditions (0.1 M HCl) using porcine gastric mucosa pepsin (Sigma-Aldrich, USA). Digestion was conducted for 48 h under constant stirring at room temperature before neutralizing the gel with 1 M sodium hydroxide (NaOH; Sigma-Aldrich, USA). The resulting hpcECM-gel was stored at 7 °C before further use.

SF solution was prepared using our previously established procedure [33, 34]. Briefly, *Bombyx mori* cocoons were degummed to remove sericin protein by boiling 10 g of cut cocoons in 4 L of 0.02 M sodium carbonate solution for 60 min and rinsing three times in deionized (DI) water. Degummed fibers were dried for 48 h and solubilized in 9.3 M lithium bromide (LiBr) solution at a concentration of 20% (w/v) for 4 h at 60 °C. The solution was then dialyzed against distilled water using regenerated cellulose dialysis tubing (3.5 kilodalton (kD) molecular weight cutoff (MWCO), Spectrum Labs, USA). Dialysis water was changed 6 times over 3 days and the resulting solution was centrifuged 2 times at 9000 rpm at 4 °C for 20 min to remove insoluble particles. The concentration of silk solution was determined by weighing a known volume of sample before and after drying overnight at 60 °C.

G-TA was synthesized via carbodiimide-mediated reaction as described previously [35]. Briefly, 2% (w/v) gelatin solutions were prepared in 0.05 M 2-(N-Morpholino) ethanesulfonic acid (MES) buffer (pH 6.0) and reacted with tyramine hydrochloride (Sigma-Aldrich, Austria) in the presence of 1-ethyl-3-(-3-dimethylaminopropyl) carbodiimide hydrochloride (EDC) (Sigma-Aldrich, USA) (184 mg per 1 g protein) and N-hydroxysuccinimide (NHS) (Sigma-Aldrich, USA) (57 mg per 1 g protein) under stirring at room temperature (24 °C) for 18 h. Solutions were dialyzed against distilled water using 3.5 kD MWCO tubing (Spectrum Labs) with 6 changes over 3 days. G-TA solution was subsequently lyophilized and stored at − 20 °C until needed.

### Hydrogel preparation and gelation kinetics

Composite hydrogels were mixed from aqueous SF, G-TA, and hpcECM solutions at a final protein concentration of 4% (40 mg/mL) in HD50 buffer (40 mM hydroxyethyl)piperazine-1-ethane- sulfonic acid (HEPES)) (Sigma-Aldrich, USA), 50 mM NaCl, and 5% dextrose at pH 7.4. SF concentration was always set to 2% and supplemented with the additional gel components (G-TA and hpcECM) to add up to a final protein concentration of 4%. Prior to use Silk and G-TA stock solutions of 6% (w/v), HRP, and H2O2 solutions were sterile filtered using Millex-GV 0.22 μm polyvinylidene fluoride (PVDF) syringe-driven filter units (Millipore, Darmstadt, Germany).

Gelation kinetics of the hydrogels were monitored at 37 °C by measuring fluorescence emission using a SpectraMax M2 multi-mode microplate reader (Molecular Devices, Sunnyvale, CA). Gelation of 150 μL solutions was initiated with 5 U/mL HRP (type VI, Sigma-Aldrich, USA) and 0.01 wt% H_2_O_2_ (Sigma-Aldrich, USA) in a black 96-well plate and fluorescence em/ex 415/315 nm every minute for 120 min. Samples were normalized to a blank measurement taken before H_2_O_2_ was added (*n* = 5).

### Rheology

Rheological properties of the hydrogel blends were measured at 37 °C using an ARES-LS2 rheometer (TA Instruments, New Castle, DE) with a 25 mm stainless steel upper parallel plate and temperature-controlled Peltier bottom plate. A 420 μL aliquot of pre-hydrogel solutions with 10 U/mL HRP was loaded onto the Peltier and the cone was lowered to 47 μm. To initiate gelation, 4.2 μL of 1% or 0.5% H_2_O_2_ was injected into the gap during a 10 s (s) pre-cycle at a steady shear rate of 100/s. The gap was encased in a humidity chamber to prevent evaporation during analysis. A dynamic time sweep was performed at 1 Hz with a 1% applied strain for 60 min to determine gelation kinetics and storage moduli. Rheological properties were measured in the linear viscoelastic region, where the storage modulus was independent of the applied strain.

### Hydrogel disc preparation

Hydrogel discs (4 mm height) were prepared with 300 μL solutions in 10 mm diameter polydimethylsiloxane (PDMS) molds. Mixtures were performed according to Table 1. Hydrogels were incubated for 90 min at 37 °C before they were overlayed with PBS buffer, or culture media when loaded with cells, and incubated for 1, 7, 14, 21 and 28 days (*n* = 4). The experiment was repeated three times resulting in a total of 12 specimens per group (*n* = 12). At the given time points four specimens of each condition were collected using a 5 mm biopsy punch and transferred to a well plate into fresh PBS solution. The resulting hydrogel cylinders with 5 mm diameter and 4 mm in height were used to perform unconfined compression tests, scanning electron microscopy (SEM) for structural analysis, and infrared spectroscopy to determine secondary protein folding.

**Table1:**
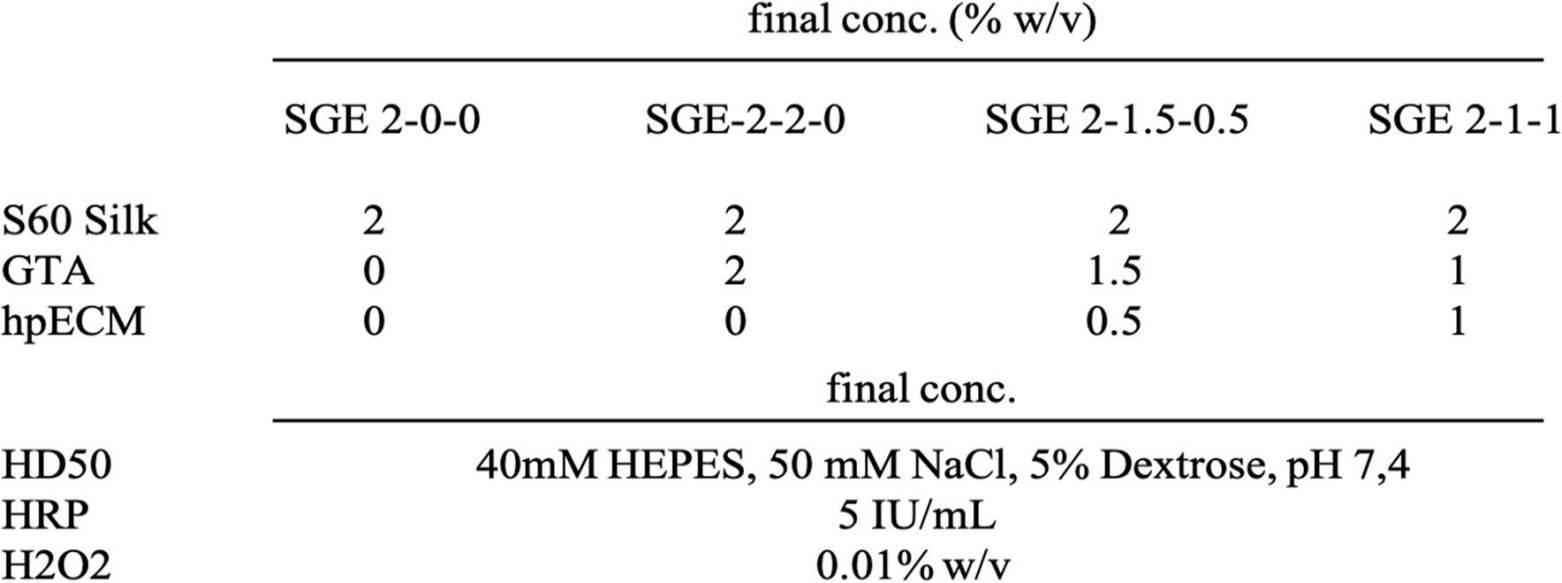
SGE hydrogel formulations

### Dynamic mechanical analysis (DMA) – compression tests

Unconfined compression was performed using an Instron 3366 Uniaxial Tensile Testing System (Instron, Norwood, MA) with a 10 N load cell. Hydrogel discs (5 mm interior diameter/4 mm height) were placed between stainless steel parallel plates and stress response and elastic recovery were monitored during 30% strain at a rate of 0.667% s^-1^. Non-stiffened samples were objected to one pre-cycle at the same strain rate to eliminate artifacts.

### Fourier transform infrared spectroscopy (FTIR)

Secondary structure of the hydrogels was analyzed using a JASCO FTIR 6200 spectrometer (JASCO, Tokyo, Japan) with a MIRacleTM attenuated total reflection (ATR) with germanium crystal as described before. After compression tests hydrogel discs were washed in deuterium oxide (Sigma-Aldrich, USA) two times for 30 min each. Measurements were conducted by averaging 32 scans with a resolution of 4 cm^-1^ between 600 and 4000 cm^-1^. *β*-sheet and alpha-helical contents were determined by analyzing the curve of the total area between 1580 and 1720 cm^-1^.

### Fluorescence cell staining and imaging

At the given timepoints, hydrogels loaded with human neonatal dermal fibroblasts (hDFN) were stained for LIVE/DEAD imaging using a 1:1000 dilution of Calcein AM (green) and Propidium iodide (red) dye (Invitrogen, Carlsbad, CA) in 10% PBS. After adding the staining solution, samples were allowed to incubate for 30 min at room temperature with shaking. To remove excess stain, samples were washed three times in PBS and then taken to the BZ-X700 Fluorescence Microscope (Keyence Corp., Itasca, IL) for imaging.

### Scanning electron microscopic (SEM) imaging

The hydrogel samples were snap-frozen in liquid nitrogen and freeze-dried for 48 h. The freeze-dried samples were broken into smaller pieces to allow a view inside the hydrogel samples. The pieces were placed on a sample holder and sputter coated with gold particles at 22 mA for 90 s before imaging with an electron microscope (Zeiss, EVO).

### In vivo, biocompatibility – subcutaneous implantation

Hydrogel discs of 8 mm diameter and 4 mm height were fabricated in autoclaved PDMS molds using 200 μL aliquots of hydrogel solutions as described in Sect. 2.4. Following 24 h of incubation in saline buffer at 37 °C, sample discs were implanted subcutaneously into 11-week-old Sprague Dawley rats (*n* = 15, 8 males and 7 females). The rats were anesthetized initially with 3% isoflurane and maintained at 2% for the duration of the surgery. Prior to making the incision, the area was shaved, sterilized three times using disinfectant and ethanol swabs, and a subcutaneous injection of the sustained-release buprenorphine (0.5 mg/mL) analgesic was administered at a dose of 1 mg/kg. Five independent incisions (6—10 mm) were made on the back of each rat to create a subcutaneous pocket using scissors and a blunt probe, and the implant was placed into this pocket. Incisions were closed with one or two stainless steel wound clips.

### Histology

To determine the inflammatory response, angiogenesis, and construct breakdown of the gels, subcutaneous explants of SGE hydrogels were collected on day 3, 30, and 90 after implantation. Samples from 5 rats were collected for each condition and time point. Recovered tissues were fixed in 4% paraformaldehyde, trimmed by standard methods and placed in cassettes for routine processing, paraffin embedding, sectioning (5 µm), and staining (hematoxylin and eosin (H&E)).

### Cell culture in hydrogels

Human dermal fibroblasts (hDFN, ATCC, Manassas, VA) were cultured in Dulbecco’s Modified Eagle Medium (DMEM)/Hams F12 high glucose supplemented with 10% fetal bovine serum (FBS), 1% non-essential amino acids (Sigma- Aldrich, USA), and 1% Penicillin–Streptomycin (Life Technologies, Carlsbad, CA). Cells between passage 4–8 were encapsulated in the hydrogels by preparing 300 μL precursor solutions of four different SGE-hydrogel blends with a final concentration of 1) silk 2% (SGE 2–0-0), 2) silk 2%, GTA 2% (SGE 2–2-0), 3) silk 2%, GTA 1.5%, hpcECM 0.5% (SGE 2–1.5–0.5) or 4) silk 2%, GTA 1%, hpcECM 1% (SGE 2–1-1). All hydrogel precursor solutions were mixed with 10 IU/ml HRP at a concentration of 8 × 10^5^ cells and concentration of 8 × 10^5^ cells/ml.

Cell-laden precursor solutions with 0.01 wt % H2O2 were allowed to cure in custom made molds for 1.5 h at 37 °C. The samples were then flooded with culture media and media was changed every 3 days.

### Metabolic activity

Metabolic activity of hydrogel-encapsulated hNDFs was measured at days 1, 3, and 7 using an alamarBlue cell viability assay (Invitrogen, Carlsbad, CA) as specified by the manufacturer. After aspirating excess culture media and rinsing with PBS, four samples of each group with cell-loaded hydrogels in 48-well plates were incubated for 3 h at 37 °C and 5% CO_2_ in 300 µl 10% alamarBlue reagent, in DMEM high glucose without phenol red (Sigma-Aldrich, USA) supplemented with 1% non-essential amino acids. After incubation, 150 μL aliquots were transferred to black 96-well plates. The remaining alamarBlue reagent was aspirated and replaced with fresh culture media to continue cell growth until the following timepoint. Fluorescence values were recorded using a microplate reader with excitation and emission set to 540 and 590 nm respectively.

### Fluorescence cell staining and imaging

At the given time points, hydrogels loaded with hNDFs were stained for LIVE/DEAD imaging. After adding the staining solution, samples were incubated for 30 min at room temperature on a shaker plate. To remove excess stain, samples were washed three times in PBS and then imaged on a BZ-X700 Fluorescence Microscope (Keyence Corp., Itasca, IL).

### 3D bioprinting

SGE 2–1-1 composite hydrogels were used as bioinks for extrusion-based 3D bioprinting using a 3D Bioplotter- Manufacturer Series (Envisiontec, G, Germany). A low temperature printhead was set to 20 °C and equipped with a 22GA sized nozzle. Pressure was set to 0.2 bar with a travel speed of 10 mm/s. Due to the low viscosity of the SGE 2–1-1 bioink, the constructs were printed into a gelatin support bath to keep the structure in shape until the matrix was fully gelled. During printing the base plate was chilled to 15 °C to keep the bath below 25 °C, where it would start melting and lose its supporting properties. The gelation was initiated by adding HRP (5 IU/mL) to the SGE 2–1-1 bioink and H_2_O_2_ (0.005%) to the gelatin slurry of the support bath. With this setup the SGE composite hydrogel begins to polymerize when both HRP and H_2_O_2_ come into contact with each other and the SF during extrusion. After printing, the support bath was directly stored at 37 °C to let the gelatin slurry melt within 15 min. After that, the slurry was washed off from the construct using prewarmed PBS buffer. The 3D printed scaffolds were then transferred to cell culture plates and incubated with cell culture media at 37 °C and 5% CO_2_ until further examination. Pilot-3D-prints were exanimated via LIVE/DEAD fluorescence staining and a metabolic activity assay on days 1, 3 and 7 after printing.

### Production of support bath

The production of a gelatin slurry support bath was published before [30]. The protocol was adapted for our needs by defining exact washing cycles and enrichment with H_2_O_2_ of the slurry to induce polymerization of the SGE hydrogels after extrusion. Briefly, 6.75 g of type B gelatin was dissolved in 150 mL of 14 mM pH 7.3 HEPES buffer solution and stirred for 5 min at 900 rpm. The gelatin slurry was heated up to 28 °C and stirred for another 30 min.

Subsequently, the solution was stored at 4 °C until the following day. Then 350 mL of cold HEPES buffer was added to the slurry and mixed with a blending machine. This solution was then refrigerated at 4 °C for an additional 24 h. On day 3, the slurry was centrifuged at 1,000 g for 3 min. The supernatant was discarded, replaced with fresh buffer to resuspend the gelatin pellet and centrifuged for another 3 min. This step was repeated two more times. At the final step the buffer was supplemented with H_2_O_2_ to reach a final concentration of 0.005% H_2_O_2_ in our gelatin slurry. After the last centrifugation step, the supernatant was discarded and the slurry was transferred into small petri dishes or well plates for FRESH printing with HRP (5 IU/mL)-loaded SGE-bioink.

### Statistical analysis

All statistical calculations were performed using Prism (GraphPad version 9.50 for Windows, Inc., San Diego, USA). Normal distribution was tested using Kolmogorov–Smirnov and D’Agos- tino-Pearson tests. Data were expressed as mean ± standard deviation. Student's t-test was used to compare means between two groups. For comparison between three or more groups one-way ANOVA with post-hoc Bonferroni testing was applied to confirm statistical significance. *P*-values below 0.05 were considered statistically significant.

## Results

### Characterization of hpcECM and G-TA

**Fig. 1:**
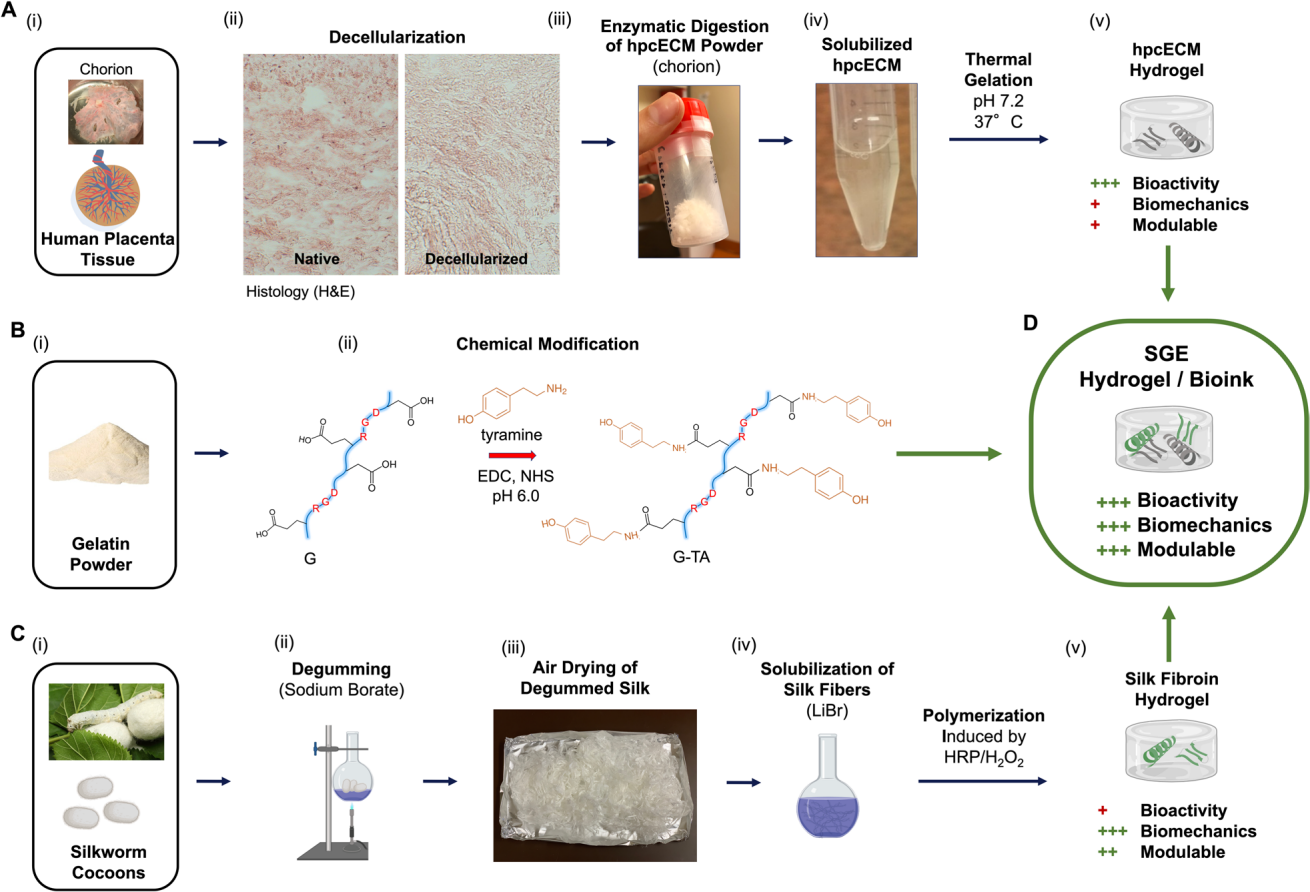
Schematic of processing the single components for the silk, tyrosinated gelatin, and human placenta ECM (SGE) composite hydrogels: **A** hpcECM hydrogel produced by tissue decellularization of chorionic tissue from the human placenta, followed by enzymatic digestion to solubilize hpcECM powder into a pre-hydrogel solution which forms a gel at physiological condition (37 °C, pH 7.2). **B** Chemical modification of bovine gelatin type B by EDC/NHS coupling of tyramine-modified amino acids to the backbone of gelatin. **C** (ii) Degumming *Bombyx mori* silk cocoon fibers using carbonate buffer (60 min boiling) followed by (iii) drying in air on aluminum foil and (iv) lithium bromide (LiBr) solubilization of silk fibers to produce a silk fibroin pre-hydrogel solution. (v) Polymerization can be chemically induced by a combination of horseradish peroxidase (HRP) and hydrogen peroxide (H_2_O_2_). **D** Mixing the three components results in SGE composite hydrogels for 3D printing and tissue engineering

**Fig. 2:**
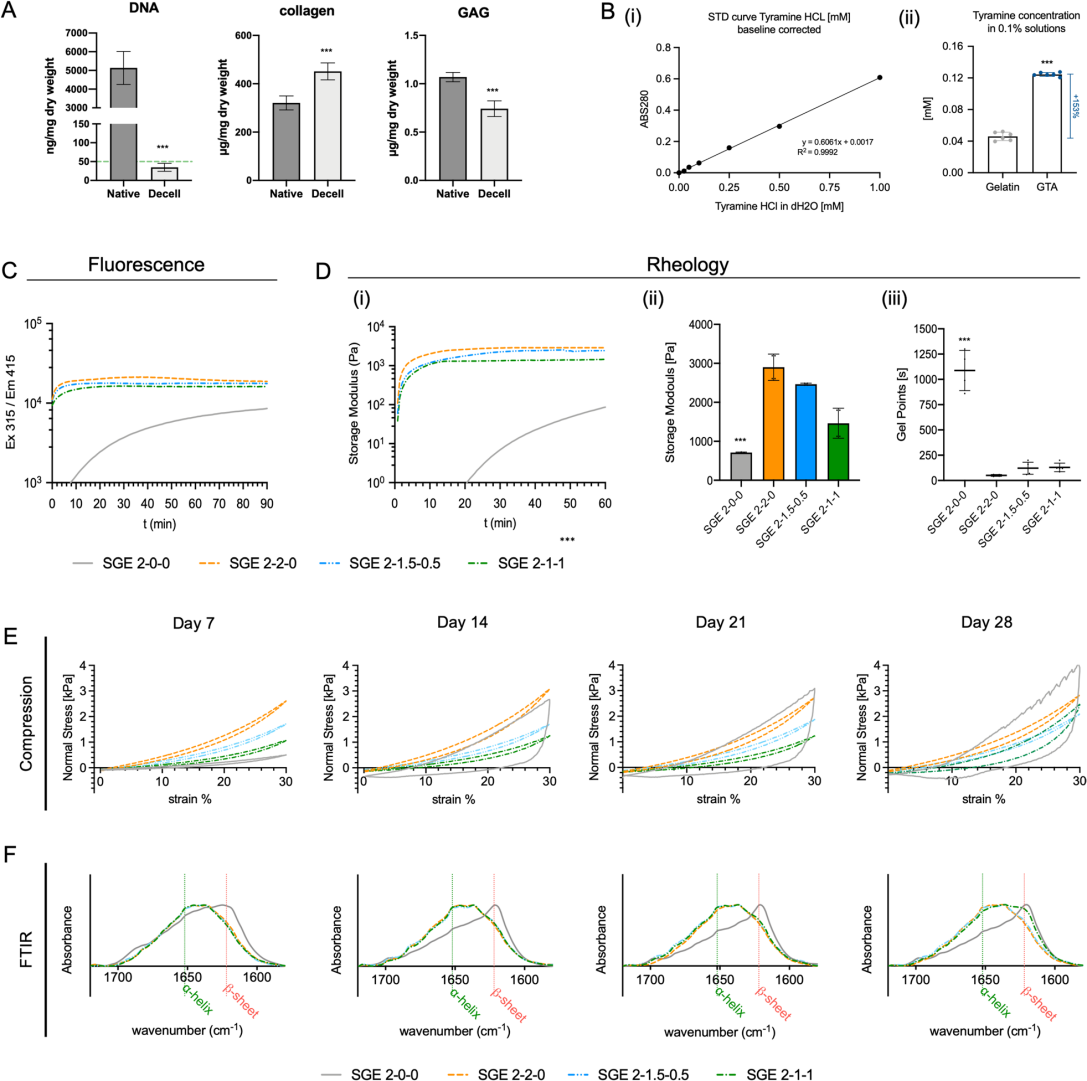
**A** biochemical assays for hpcECM characterization. Values are compared to native tissue and show the near complete removal of DNA while preserving matrix components such as collagen and glycosaminoglycans (GAGs). **B** Determination of chemical modification efficiency. Tyramine peptides were covalently bound to gelatin to create gelatin with enriched phenol groups (GTA). (i) Standard curve for photometric quantification of phenol (Abs280 nm) and (ii) determination of tyramine concentration in GTA compared to gelatin. **C**-**F** Biomechanical analysis of four SGE composite hydrogel formulations: SGE 2–0-0 (SF 2%), SGE 2–2-0 (SF 2%, GTA 2%), SGE 2–1.5–0.5 (SF 2%, GTA 1.5%, hpcECM 0.5%), SGE 2–1-1 (SF2%, GTA 1%, hpcECM 1%). **C** Kinetics of SGE hydrogel formation using fluorescence measurement. **D** Rheological analysis showing gelation time points. **E** Uniaxial compression tests and (**F**) Spectral analyses using Fourier Transformation Infrared Spectroscopy (FTIR)

H&E staining and DNA quantification confirmed the complete decellularization of placental chorionic tissue used for the preparation of hpcECM hydrogels (Fig. 1 A (ii and Fig. 2A). Decellularized tissue showed no visible nuclei and had DNA values of 34.8 ± 10.6 ng DNA / mg dry weight, which represents a reduction of 99.3% compared to native control tissue. On the other hand, substantial preservation of both collagen (native: 320.7 ± 29.1 µg/mg, decell: 451.3 ± 34.9 µg/mg dry weight) and proteoglycans and glycosaminoglycans (GAG) (native: 1.1 ± 0.1 µg/mg decell: 0.74 ± 0.01 µg/mg dry weight) was determined by biochemical quantification assays (Fig. 2A). Rapid gelation tests confirmed the temperature and pH-neutral gelation of the final hpcECM hydrogels used for blending with the SF and G-TA (Suppl. Figure 2).

Tyramine-conjugated gelatin (G-TA) was obtained by carbodiimide coupling. UV absorption analysis detected significantly increased tyramine content in G-TA hydrogels, using the absorption peak at 280 nm to quantify the phenolic compounds (Fig. 2B). Relative to our standard curve, there was a 153% increase in phenolic compounds in G-TA compared to pure gelatin.

### Gelation of composite hydrogels

Covalent cross-linking of SF only and SF, G-TA, and hpcECM (SGE) composite hydrogels was induced by HRP and H_2_O_2_ through the formation of tyrosine-tyramine linkages as described previously [27]. Crosslinking kinetics of the SGE hydrogels were monitored by quantifying the fluorescence emission and rheological measurements. For fluorescence quantification, excitation and emission wavelengths of 315 nm and 415 nm, respectively, were used. Curves of SG and SGE hydrogels were hyperbolic-like and reached a plateau significantly faster compared to curves of SF only hydrogels, which displayed a sigmoidal shaped curve that did not reach a plateau at t = 90 min, indicating that crosslinking was not completed at this timepoint. The relative intensity of fluorescence emission was found to increase significantly in G-TA containing hydrogels (Fig. 2C).

Rheological analysis confirmed these previous observations. Time dependent kinetics showed rapid gelation times in SG and SGE composite hydrogels but not in SF only hydrogels. After 60 min the shear storage moduli were strongest in SGE 2–2-0 hydrogels (2.885 ± 0.010 kPa) followed by SGE 2–1.5–0.5 (2465 ± 0.289 kPa), SGE 2–1-1 (1.440 ± 0.027 kPa) and SGE 2–0-0 (0.703 ± 0.010 kPa) hydrogels, with the lowest values not having reached the plateau at that time (Fig. 2D (I, and ii)). The gelation start and the gelling point (G”/G’ < 0.05) decreased with increasing G-TA content in the composite hydrogels. While SGE 2–0-0 hydrogels showed a gelation point above 1000 s, it took less than 200 s to reach the gelation point for G-TA containing hydrogels, and only ~ 50 s for SGE 2–2-0 composite hydrogels (Fig. 2D (iii)).

### Biomechanical properties of composite hydrogels

SGE composites were incubated in PBS at 37 °C and their compressive properties were analyzed over 4 weeks. Unconfined compression analysis showed elastic behavior in all hydrogel groups for the first week. Over time, the SGE 2–0-0 hydrogels lost their elastic behavior. From day 7 onward the SGE hydrogels became stiff and brittle, as indicated by a large hysteresis of the compressive strength curve on days 14, 21 and 28 (Fig. 2E).

The initial compression strength increases with a higher proportion of G-TA due to more cross-links of the reactive phenolic groups with the SF. The measured compressive moduli were 0.499 ± 0.080 kPa in SGE 2–0-0, 2.460 ± 0.344 in SGE 2–2-0, 1.798 ± 0.265 in SGE 2–1.5–0.5, and 1.284 ± 0.263 kPa in SGE 2–1-1 composite hydrogels on day 7 (*n* = 8). All G-TA containing composite hydrogels stayed elastic until day 21, while the composite hydrogel with the lowest G-TA concentration SGE 2–1-1 started to lose some elasticity on day 28, indicated by an increase in compressive modulus and the hysteresis of the curve. SGE 2–2-0 and SGE 2–1.5–0.5 composite hydrogels stayed elastic with constant compressive strength throughout the 4 weeks of observation (Fig. 2E, Suppl. Figure 1).

### Changes in secondary protein structures in SGE composite hydrogels

The IR absorption spectra of the SGE composite hydrogels were analyzed by ATR-FTIR to determine protein folding such as the proportion of α-helical and β-sheet structures over 4 weeks of incubation in PBS at 37 °C. The absorption peak for alpha-helical protein structures is at 1652 nm and β-sheets are detected at 1623 nm wavelength [36, 37]. An overlay of the FTIR spectra in the waveband between 1578 and 1720 nm allowed comparisons between the proportions of the different protein structures in the SGE hydrogels to determine the degree of crystallization (Fig. 2F). SF only hydrogels, SGE 2–0-0, showed a high fraction of β-sheets from day 7, in contrast to the other groups that contained various concentrations of G.TA. Significant crystallization in SGE 2–0-0 hydrogels appeared from day 14 onwards, with only minor signals for α-helical structures and a prominent peak for β-sheet structures.

The time course of the changes in the proportions of the protein structures was congruent with the results of the biomechanical measurements. SGE-2–2-0 and SGE 2–1.5–0.5 remained stable for the entire 4 weeks with low β-sheet formation and prominent α-helical structure fraction. SGE 2–1-1 stayed stable for the first 3 weeks and showed an increased β-sheet content at the final time point on day 28. However, in contrast to SF only hydrogels, the α-helical content in SGE 2–1-1 composite hydrogels remained high even on day 28.

### Appearance and structural analysis with SEM

**Fig. 3:**
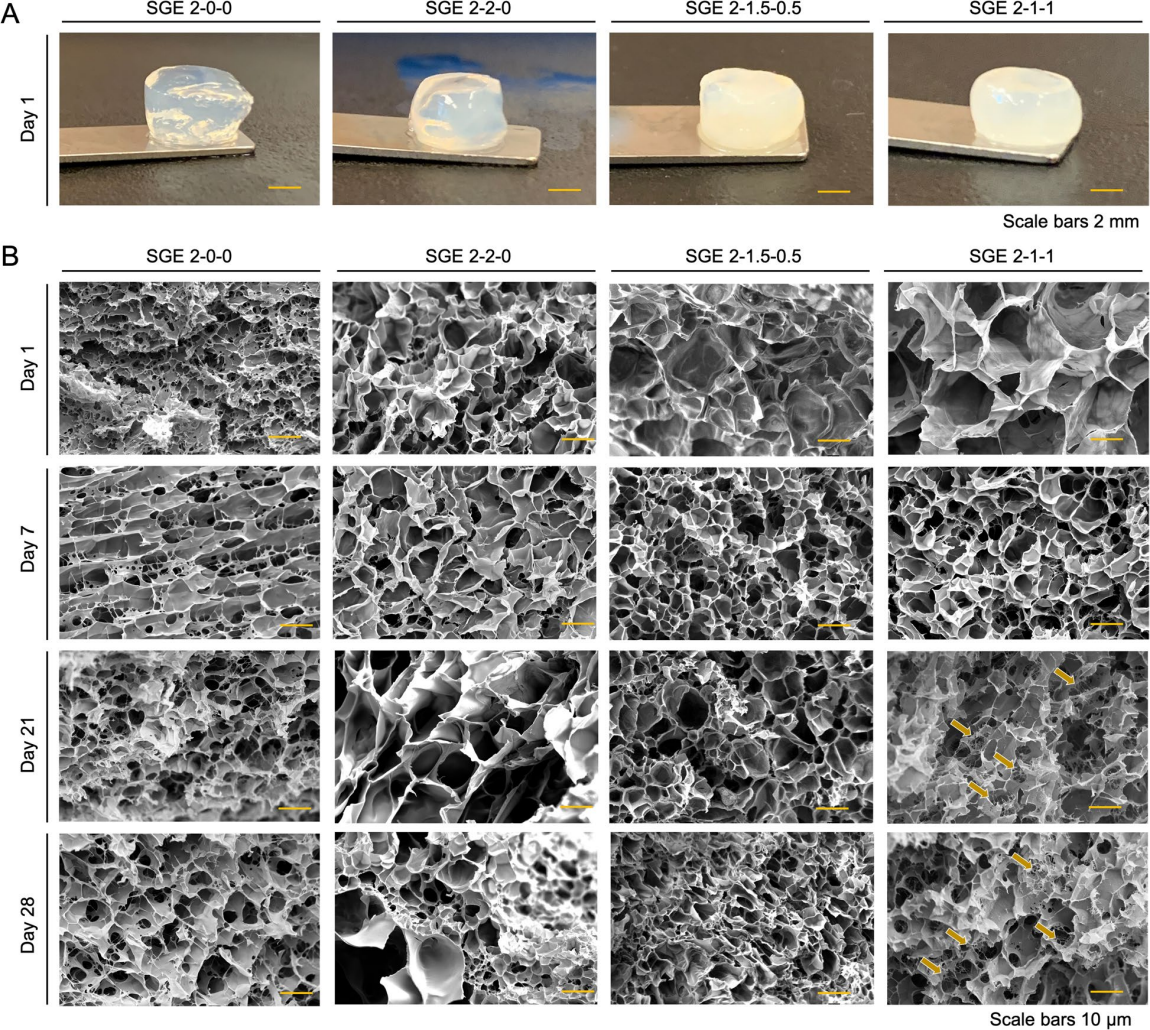
**A** Polymerized cylindrical SGE hydrogels SGE 2–0-0 (SF 2%), SGE 2–2-0 (SF 2%, GTA 2%), SGE 2–1.5–0.5 (SF 2%, GTA 1.5%, hpcECM 0.5%), SGE 2–1-1 (SF2%, GTA 1%, hpcECM 1%). with changes in transparency according to composition. **B** Surface structure analysis by scanning electron microscopic imaging of SGE composite hydrogels chemically crosslinked by HRP/H_2_O_2_ and incubated in PBS up to 28 days. Arrows in SGE-2–1-1 group show hpcECM deposition by forming fibers across the pores. (Scale bars 50 µm)

Unlike transparent SF-only hydrogels, the composite hydrogels were opaque, and the opacity increased with increasing G-TA and hpcECM content (Fig. 3A). Scanning electron microscopy images of SGE hydrogels at various time points showed porous structures (Fig. 3B). The addition of G-TA led to larger pores that were more rounded compared to SGE 2–0-0 hydrogels which contained SF only. SGE 2–2-0 and SGE 2–1.5–0.5 composite hydrogels remained the most consistent in topography and shape of the pores over 28 days. SGE 2–1-1 composite hydrogels lost structural properties from day 14 onward, with the formation of additional fibrous structures between the pores (indicated by arrows).

### In vivo – biocompatibility, cell migration, and remodeling

**Fig. 4:**
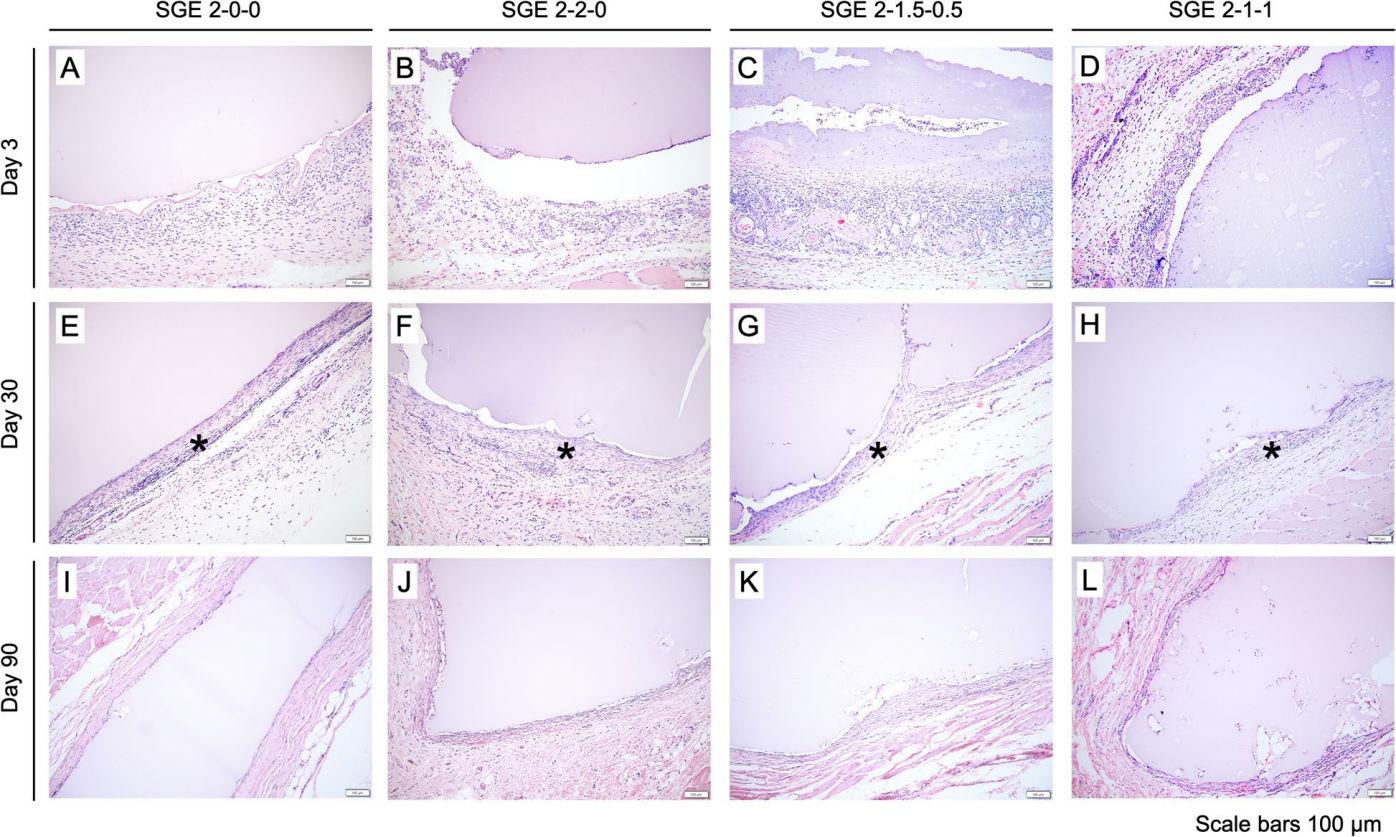
Histological examination of subcutaneous implanted SGE hydrogels using H&E staining reveals low immunogenic potential of all samples. Day 30 samples **E**–**H** contained scattered to diffuse low density infiltrates of macrophages, and lymphocytes in a thin band of fibrosis (*) surround the implant. By day 90 (I-L), there is little evidence of inflammation due to lack of granulocyte invasion in the surrounding tissue

The four SGE composite hydrogel constructs had similar morphology and tissue response compared to each other at days 3, 30, and 90 post-implantation. These time points allowed for a detailed understanding of the long-term stability and biocompatibility of silk-based scaffolds in animals. Histological results indicated a typical and mild foreign body response (Fig. 4). At day 3, the subcutaneous implants had a low-density of infiltrated macrophages, lymphocytes, and neutrophils and prominent small caliber blood vessels (neovascularization) surrounding the implant. Day 30 samples contained scattered to diffuse low density infiltrates of macrophages and lymphocytes in a thin band of fibrous tissue surrounding the implant. Of the implants that remained at 90 days, only scattered mononuclear cells remained with a thin fibrous capsule around the implants.

### In vitro—cell growth within SGE composite hydrogels

**Fig. 5:**
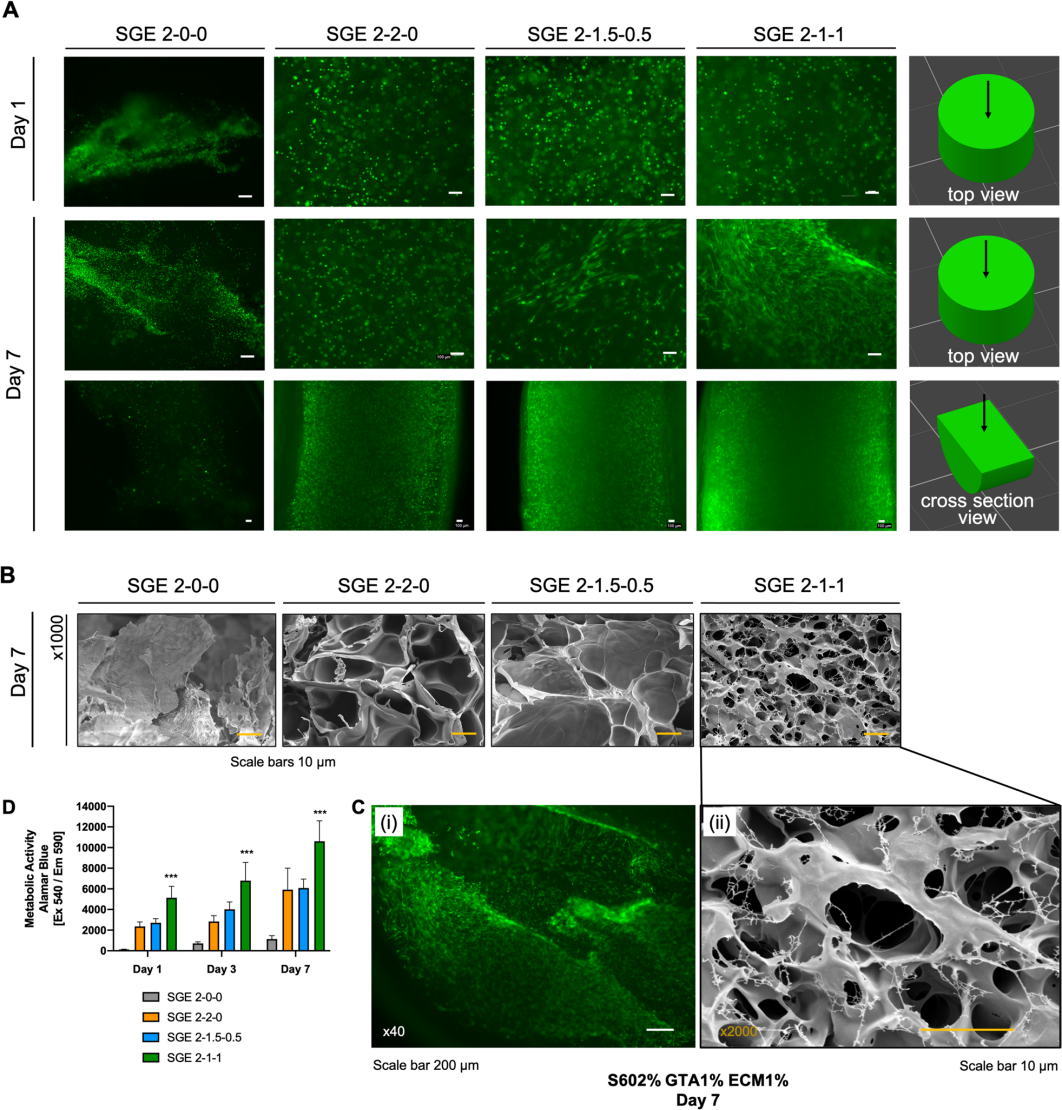
SGE composite hydrogels containing human fibroblasts (hNDF) and polymerized in cylindrical casts, stained for living cells using calcein AM dye. **A** Cell imaging at an early (day 1) and late (day 7) time point. On day 7, cylindrical scaffolds were cut in the middle to show cell distributions. **B**, **C** Scanning electron microscopy on day 7 for confirmation of fibroblasts stretching over the SGE 2–1-1 composite hydrogel. Within all other groups, no cells were detected by this method. **C** (i) Calcein-AM staining of human neonatal fibroblasts encapsulated at SGE 2–1-1 hydrogels at day 7. **D** Metabolic activity measured by Alamar Blue showed low metabolic signals of human neonatal fibroblasts in SF only and moderate metabolic signals in the SGE 2–2-0 and SGE 2–1.5–0.5 gels. The highest metabolic activity was measured in SGE 2–1-1 composite hydrogels compared to all other groups. Data are shown as mean ± SD. *** indicates significant differences of p < 0.05 between SGE 2–1-1 and all other groups

SGE composite hydrogels were loaded with hDFN and filled into cylindric casts for gelation. Hydrogels were monitored for cell distribution, morphology and metabolic activity (Fig. 5). SGE 2–0-0 hydrogels showed unevenly distributed live cells in the form of small spheres, indicating a lack of adhesion to the SF matrix. All other SGE composite hydrogels at day 1 showed a homogenous distribution of the cells, which remained rounded at this time point. After one week of incubation, the cells started to spread out and change morphology, particularly in the SGE composite hydrogels containing hpcECM (SGE 2–1.5–0.5 and SGE 2–1-1). Cross-sectional views of the cylindrically-shaped hydrogels showed living cells homogenously distributed throughout the entire matrix (Fig. 5A). Composite hydrogels with the highest concentration of hpcECM, SGE 2–1-1, most effectively supported spreading and natural morphology of the cells. SEM analysis clearly showed that the fibroblasts in the SGE 2–1-1 samples were stretched over the matrix surface. In contrast, such conditions were not observed in the other groups (*n* = 3) (Fig. 5B, C). Metabolic activity was also determined to be significantly higher in cells seeded in SGE 2–1-1 composite hydrogels (Fig. 5D).

### Printability and cell survival in SGE 2–1-1 bioink

**Fig. 6:**
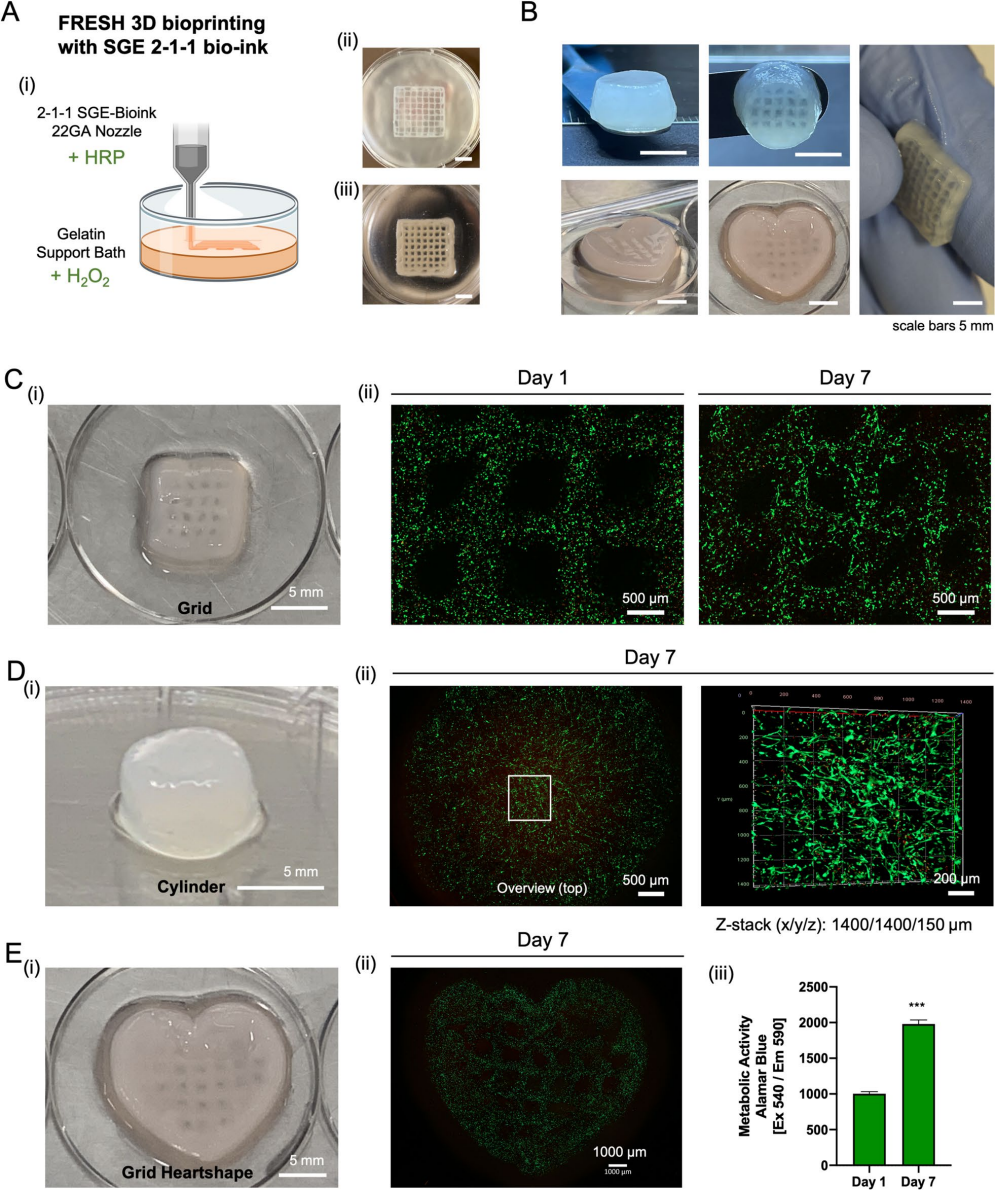
FRESH bioprinting using SGE 2–1-1 bioink loaded with human neonatal fibroblasts. **A** (i) Drawing of the printing setup with addition of the crosslinking agents to the ink (+ HRP) and the support bath (H_2_O_2_). (ii) Printed grid casted into the support bath and (iii) the same grid after washing away the support bath. **B** Images of different shapes printed with SGE 2–1-1 **C**-**E** (i) Images of 3D bioprinted shapes (**C** (ii)) Live/Dead staining of encapsulated cells into a grid (8 × 8 mm) on day 1 and day 7. **D** (ii) Live/dead staining on day 7 shows high cell viability (top view) throughout the construct in a z-stack from the middle area of the cylindrically shaped scaffold (**E** (ii)) Day 7 LIVE/DEAD staining of a heart shaped grid (iii). Alamar Blue staining of the printed grid showed significantly increasing metabolic activity over time (*p* < 0.05)

The SGE 2–1-1 hydrogels showed the best cell–matrix interactions in our experimental setup for testing cell incorporation into the matrix. Therefore, this formulation was used as a bioink for a pilot test on general printability and cell viability when printing constructs loaded with hDFN (Fig. 6). SGE 2–1-1 bioink was loaded into a syringe and mounted on a pneumatic printing system using the low temperature head of an Envisiontec Bioplotter. The bioink could be steadily extruded through a 22 GA (gauge) nozzle, allowing the controlled deposition of 400 μm diameter filaments with a commercial bioprinter using pneumatic extrusion. Test prints and the final printing setup used for SGE 2–1-1 bioink can be seen in supplemental material (Suppl. Figure 4 & 5). These filaments could be overlaid or stacked to form 3D constructs while maintaining high cell viability of the encapsulated cells. Due to the low viscosity of this bioink, biomaterial constructs were printed with a pressure range between 0.1–0.2 bar into a gelatin support bath. Our gelation strategy for 3D printing with SF-containing bio-inks, in which we mix the bioink with HRP and enrich the support bath with H_2_O_2_, enabled us to print complex constructs of different shapes. Gelation occurs rapidly therefore the samples can be incubated at 37 °C for 25 min to melt the gelatin bath immediately after printing. Rectangular grids and cylinders were printed for initial test prints. The constructs were dimensionally stable and showed promising haptic properties during handling (Suppl. Video). In a second step, various shapes as grids or cylinders were printed with hDFN preloaded into the bio-ink. LIVE/DEAD staining on day 1, 3, and 7 showed high cell viability and early cell stretching along the matrix.

## Discussion

In this study, a 3D bioprintable silk, gelatin, and human placental ECM composite hydrogel was developed to design tailorable cell-laden constructs in soft tissue engineering. When further evaluated as a bioink, this material provided a mechanically stable 3D elastic matrix supporting the encapsulation and proliferation of cells throughout the 3D printed constructs.

The principal focus of soft tissue engineering is to generate a complex 3D functional soft construct derived from living cells. Two major components reconciling biological and engineering performance in this process are biomaterial selection and the manufacturing technology utilized. However, for translational application, there are some critical measures to fulfil, such as tuning the mechanical properties of the biomaterial to match the target in vivo biological environment; ensuring biocompatibility that supports cell migration, ECM production, and long-term exchange of oxygen and nutrition; and ultimately the availability of easy-to-use technology creating 3D constructs with high shape fidelity to the target tissue.

Silk fibroin’s unique repetitive protein sequences provide exceptional structural and mechanical stability in a biomaterial [19, 38]. However, SF hydrogels are less bioactive and they can become brittle over time due to the accumulation of β-sheet structures of the peptides. Therefore, this biomaterial has been used as a composite with a range of other natural (collagen, gelatin, elastin, chitosan, hyaluronic) [39–41] and synthetic (polystyrene, polyurethane and poly (L-lactide)) polymers [42, 43] in order to enhance the functionality and extend the application of silk-based biomaterials. In general, blending silk with other polymers leads to improvements in some of the critical criteria/measures mentioned above. Natural polymers combined with silk are particularly effective for increasing cell responses. For instance, silk-chitosan blends increased fibroblast cell proliferation while preserving compressive strength. In addition, collagen/silk fibroin composites with PLGA microspheres supported bone marrow stromal cell (BMSCs) proliferation, leading to enhancements in articular cartilage regeneration [44, 45].

Gelatin has been commonly used as a composite material in silk-based constructs in bone [46] and cartilage [47] hard tissue engineering, and in different scaffold formulations such as meshes [48] or microcarriers. The feasibility of silk fibroin/gelatin microcarrier scaffolds for bone tissue engineering were demonstrated [49] and gelatin improved cell adhesion and viability within the microcarriers. However, this method utilized solvents and acids, potentially affecting cytocompatibility. The development of cross-linker-free 3D printed mesh scaffolds was demonstrated [50], where the bioink was printable without crosslinker to avoid cytocompatibility issues. Shape fidelity immediately after printing was high. However, the stability of the construct significantly decreased due to the high swelling ratio, and complete degradation was observed within 28 days. In a recent study, a macroporous SF-gelatin bioink was developed [51]. The bioink had a high gelatin content, overcoming the crystallization and stiffness drawbacks. The new ink was cytocompatible both in vitro and in vivo. However, the cells were not encapsulated throughout the construct in order to support 3D regeneration; rather, cells were seeded on the surface of the construct after printing. This was a positive initial proof-of-concept step that showed the cyto- and bio-compatibility of gelatin-augmented inks, but did not extend to bioprinting cell-laden matrix grafts.

In our previous work, silk-gelatin composites delayed crystallization and preserved elastic behavior [27]. However, the bioactivity was not satisfactory, as cells did not spread throughout the constructs, suggesting a need to further improve cell migration and cytoskeletal rearrangement. Therefore, we sought to further improve cell interactions with silk-based hydrogels, and focused on enhancing biological functionality while preserving the desirable mechanical stability.

In the current study, SF/G-TA hydrogels were mixed with human placental chorion ECM (hpcECM) to increase bioactivity. As a clinical waste product after birth, the human placenta represents an excellent source of healthy perinatal-derived human tissue that is ethically safe and has high biological activity [52, 53]. The placenta is mainly formed by the cells of the embryo’s blastocyst. By selectively using chorionic plate parts, we ensure that only material of fetal origin is processed while achieving a high yield. For the production of the placenta ECM, tissues from nine different donors were pooled into one batch. This overcomes possible donor variations which could alter the function of this natural biomaterial. The decellularized ECM was tested for sufficient reduction of cellular components and preservation of matrix proteins such as collagen and proteoglycans (Fig. 2A). The decellularization process was designed to leave only a maximum residual amount of DNA, less than 50 ng/mg tissue dry weight, which prevents potential host tissue immune responses [54].

Since the natural ECM contains glycosaminoglycans with high binding activity [31], initially we hypothesized that mixing SF with hpcECM alone could cause a delay in crystallization of the SF hydrogels. However, the initial experiments showed that depending on the ratio of the hpcECM to SF hydrogels, only the overall topography of the scaffolds varied, not the crystallization process (Suppl. Figure 1). The β-sheet content increased in these hydrogels over time and they became embrittled after 7 days of incubation. Therefore, we proceeded with the use of tyramine enriched gelatin (G-TA) in addition to hpcECM to simultaneously hinder silk crystallization and enhance bioactivity.

The production of the SF hydrogels was designed to meet certain criteria. Degumming time affects the properties of the silk material, including average fragment size, strength within materials and degradation rate [55]. Most commonly, degumming times varies between 30–90 min, with the longer degumming time resulting in smaller silk fragments. This reduces the viscosity of the starting material as well as the stability of the cured construct. The degradability of the material increases with prolonged boiling time [56, 57]. Our hypothesis was that with a degumming time of 60 min and a constant SF concentration of 2% (w/v) we could generate silk-based hydrogels suitable for soft tissue fabrication based on mechanical stability, printability, and degradation rate, which is consistent with the silk percentages reported elsewhere [58].

The total protein concentration of the SGE composite hydrogel mixtures was 4% (w/v). All optimized formulations had a final concentration of 2% low molecular weight SF (60 min boiled). The 2% SF (w/v) was mixed with either G-TA only or a combination of G-TA and hpcECM to generate the SGE hydrogelsSF mainly provides the mechanics in the constructs; control samples containing just SF (SGE 2–0-0) had a total protein concentration of 2% (w/v).

Classical SF crystallization in SGE hydrogels was actively delayed when mixed with G-TA. Moreover, when a certain concentration of hpcECM was added, sufficient cell adhesion points were present to support the spreading and movement of human fibroblasts encapsulated in these composite hydrogels. There were two critical measures to ensure the best functionality of these SGE composite hydrogels. First, stable formation of ECM networks in composite hydrogels seems to occur only at concentrations above about 1%. Therefore, the final hpcECM concentration should be adjusted to or above this threshold. This conclusion is supported by the fact that SGE 2–1-1 hydrogels had the best cell spreading and significantly highest metabolic activity of all groups (Fig. 5A,C,D, Suppl. Figure 3). Only a moderate improvement in cell behavior was observed for hydrogels with lower hpcECM concentrations (SGE 2–1.5–0.5) compared to the SGE 2–2-0 hydrogels without hpcECM. SEM imaging further confirmed this observation. Hydrogel fibers/fibrils were detected only in the SGE 2–1-1 group and not in SGE 2–1.5–0.5 samples. Additionally, when gelation of hpcECM samples with different concentrations was evaluated, only formulations containing hpcECM concentrations of 1% and above gelled (Suppl. Figure 2).

The second critical point when using a SGE formulation was the G-TA concentration. The results suggested that a minimum concentration of G-TA should be 1.5% if complete avoidance of β-sheet structure formation over time is the goal. In general, viscoelastic materials cannot store 100% of the energy under deformation and they lose part of this energy. This loss energy is also referred to as hysteresis loss [59]. When the elasticity of a hydrogel decreases, the material becomes stiffer. This can be observed by a higher hysteresis loss, indicated by a larger area between the two curves (load and unload) (Fig. 2E). Hydrogels containing only SF (SGE 2–0-0) lost their elasticity after a few days and showed a large hysteresis and high content of β-sheet structures with a concomitant low number of α-helical structures (Fig. 2E & F). In contrast, samples with at least 1.5% GTA content or higher showed consistently low values for β-sheet structures and high values for α-helical structures for the entire observation period of up to 28 days. SGE 2–1-1 hydrogels with a 2:1 (SF: G-TA) ratio, showed complete hinderance of crystallization for the first 21 days. However, the level of β-sheets increased at the final time point (after 28 days) while levels of α-helical structures remained high, resulting in the observed elastic behavior (Fig. 2F). Haptic properties and compressive tests further confirmed the elastic behavior and stability of the SGE 2–1-1 hydrogels despite the slight increase in β-sheet content at day 28. This finding highlighted that the stability of the α-helical structures over time could counteract the stiffening of the gels. This hypothesis will be an interesting consideration in future investigations to elucidate the interplay between the α-helical and β-sheets structures in order to maintain elasticity while limiting β-sheet content.

Cell based experiments confirmed a significant elevation in metabolic bioactivity of hDFN cells in SGE 2–1-1 composite hydrogels compared to the other groups. Cross-sectional and surface immune florescence imaging of this hydrogel construct identified a dense and evenly-distributed population of elongated cells, indicating support by the hydrogel for cell migration and cytoskeletal rearrangement. This observation was also present in the SGE 2–1.5–0.5 composite, but in a lower intensity. All of these results highlighted the potential of these hpcECM hydrogels in supporting cell adhesion and proliferation with high binding affinity (Fig. 5). Furthermore, the in vivo subcutaneous implantation of all composite hydrogels exhibited no excessive inflammatory reaction in both early and late phases of implantation. A very thin fibrous tissue containing low densities of macrophages and lymphocytes further confirmed the biocompatibility and stability of the gels for 90 days in vivo. Cell-free SGE hydrogels were transplanted subcutaneously into rats. Our results show that the materials used have good biocompatibility. However, we see limited cell migration and associated degradation of the material. This could be changed by loading the SGE hydrogels with growth factors or by loading the 3D constructs with autologous cells before implantation. Furthermore, the degradation of the SGE can also be modified by changes in the chemical processing of the silk. The findings from this paper will be used to investigate issues related to the maturation of 3D bioprinted SGE scaffolds in further studies. Bioinks are essential but challenging components in 3D bioprinting. Along with extrudability, they provide a soft environment for cells, creating a mechanically stable complex structure that mimics the target human tissues. In this study, we decided to use a commercial printing system with pneumatic extrusion which is the most common technology in 3D bioprinting. We selected a nozzle size of 400 µm in order to print in a way that is gentle to the cells. A reduction of the extrusion thickness and its direct effect on different cell types will be addressed in further studies. In general, initial test prints have shown that extrusion down to 100 µm diameter fibers is possible from the materials developed here. For even more accurate printing with such a low-viscosity bioink, we suggest mechanical extrusion technology as we have previously shown [18, 60]. Due to low-viscosity of the bio-ink the FRESH printing method was optimized based on our need to utilize SGE 2–1-1 composite hydrogels as a bioink. HRP and H_2_O_2_ were utilized as chemical/enzymatic crosslinkers and were kept spatially separate until extrusion. This was done by mixing the HRP with the SGE bioink while supplementing the support bath with the H_2_O_2_. The crosslinkers were mixed only during extrusion, avoiding early polymerization of the SGE bioink. Our homemade gelatin bath has been shown to be stable for several hours at a room temperature of 20–25 °C without losing its physical support during printing. Instant polymerization immediately after extrusion permits the incubation of the bath after printing. Short residence times of the cells during the printing process has a positive effect on the cellular and biological components of constructs. After a relatively short incubation time (maximum 20 min) the final printed construct could be extracted from the bath and transferred to cell culture media. The printed constructs with various shape were stable and effectively supported cell proliferation and migration (Fig. 5).

In this study the development of a novel silk-gelatin-ECM based bioink formulation is shown that was printable with an optimized FRESH printing technology. The use of H_2_O_2_ to crosslink polymers can have cytotoxic effects even at low concentrations of 0.1 to 10 mM [61]. However, it has been observed that rapid consumption of H_2_O_2_ via HRP-mediated oxidative crosslinking reactions are attenuated [62]. In our novel 3D bioprinting combination of FRESH and HRP/H2O2, the H_2_O_2_ is in the support bath and the HRP is mixed with the cells in the bioink. Therfore, HRP-induced crosslinking occurs immediately upon contact of the bioink with the support bath during printing. High cell viabilities in our test prints with H_2_O_2_ concentrations between 0.001—0.0001% indicated that H_2_O_2_ was efficiently consumed by HRP during printing.

The resulting cell laden 3D printed hydrogel constructs possess excellent potential for applications in soft tissue repair and regeneration. The microporous structure of the SGE 2–1-1 formulation containing 1% hpcECM supported sufficient nutrients, gas, and waste byproduct interchange throughout the hydrogel matrix, enabling cell migration, proliferation, and differentiation. These constructs were bioactive and biocompatible with adequate biomechanical properties and an absence of necrosis over time.

## Conclusions 

The data presented here revealed the potential of SGE composite hydrogels for soft tissue engineering with silk, tyramine enriched gelatin, and human placental ECM. The spontaneous process of silk crystallization was decreased and we were able to observe increased metabolic activity and cell spreading within a soft silk composite mixed with placental ECM. This system elevates the potential for using silk for soft tissue applications. To our knowledge, a silk and hpcECM-based bioink has never been used in FRESH 3D bioprinting before. With this newly established method, it will be possible to print highly complex silk scaffolds with or without cell loading in the future. For instance, in future studies the bioink and printing set-up will be used to print cardiac muscle-like tissue by printing constructs of the SGE material mixed with cardiomyocytes and cardiac fibroblasts.

### Supplementary Information

**Additional file 1: Figure S1.** (A) SF hydrogels begin to crystallize over time by forming βsheet structures. (i) FTIR (ii) peak deconvolution determining β-sheet content. (B) SEM of SF mixed with two different concentrations of hpcECM to show structural changes. (C) hpcECM did not delay crystallization of SF hydrogels shown by (i) FTIR and (ii) by calculation of the increased β-sheet content after 7 days.

**Additional file 2: Figure S2.** For rapid gelation, 200 µl of the pre-gel was added to a 1.5 ml reaction tube and incubated at 37°C. Using hpcECM pre-gel solutions at various low concentrations from 0.1% to 2% (w/v) showed that gelation only started completely at 1% (w/v).

**Additional file 3: Figure S3.** Statistical analysis of the metabolic activity of human fibroblasts encapsulated at SGE hydrogels showed significant increase in cell activity in SGE 2-1-1 hydrogels compared to all other groups. SF only (SGE 2-0-0) hydrogels showed the significantly lowest cell activity.

**Additional file 4: Figure S4.** Test prints to define: (A) print settings as pressure (0.3 bar) and nozzle speed (12 mm/s) while printing and (B) define H_2_O_2_ concentration in the support bath, changing H_2_O_2_ concentrations in the support bath and comparing gelation of the 3D scaffold. H_2_O_2_ concentration which should be as low as possible to protect cells from damage during printing, choosing 0.0005% final concentration in the support bath.

**Additional file 5: Figure S5.** Final printing setup used for SGE 2-1-1 bioink.

**Additional file 6: Video.** Haptic test with 3D bioprinted grid.

## Data Availability

The datasets used and/or analysed during the current study are available from the corresponding author on reasonable request.

## Abbreviations

ATR: Attenuated total reflection
DI: Deionized water
DMEM: Dulbecco’s Modified Eagle Medium
EDC: 1-ethyl-3-(-3-dimethylaminopropyl) carbodiimide hydrochloride
FBS: Fetal bovine serum
FRESH: Free reversible embedding of suspended hydrogels
FTIR: Fourier transform infrared spectroscopy
GTA: Tyramine-substituted gelatin
H&E: hematoxylin and eosin
H2O2: Hydrogen peroxide
HCl: Hydrochloric acid
HEPES: Hydroxyethyl)piperazine-1-ethane- sulfonic acid
hDFN: Human dermal fibroblasts, neonatal
hpcECM: Human placenta chorion extracellular matrix
hpECM: Human placenta extracellular matrix
HRP: Horseradish peroxidase
IU: International-Units
kD: Kilodalton
LiBr: Lithium bromide
MES: 2-(N-Morpholino) ethanesulfonic acid
MWCO: Molecular weight cutt off
NHS: N-hydroxysuccinimide
PBS: Phosphate-buffered saline
PDMS: Polydimethylsiloxane
PEG: Polyethylene glycol
PHEMA: Poly(2-hydroxyethyl methacrylate)
PLA: Polylactic acid
S60: 60 minutes boiled silk
SF: Silk fibroin
SGE: Silk - Gelatin - ECM
