# Supplementary figures and images for: Silk fibroin, gelatin, and human placenta extracellular matrix-based composite hydrogels for 3D bioprinting and soft tissue engineering
Source: Biomater Res. 2023 Nov 17;27:117. doi: 10.1186/s40824-023-00431-5 (PMC10656895; doi:10.1186/s40824-023-00431-5)

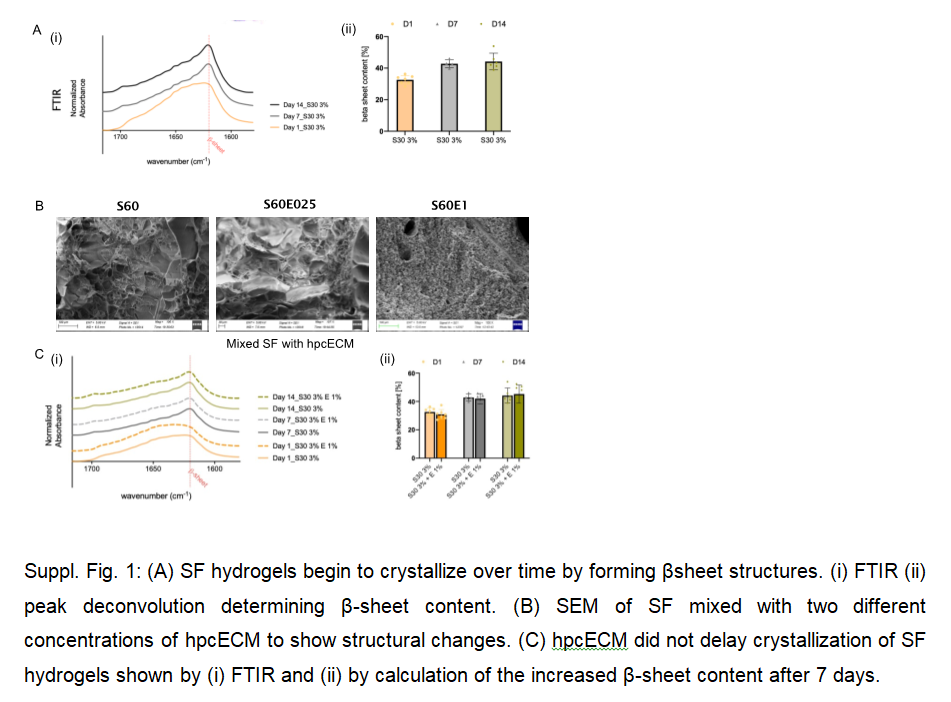

Supplement: Supplementary file 1 — Additional file 1: Figure S1. (A) SF hydrogels begin to crystallize over time by forming βsheet structures. (i) FTIR (ii) peak deconvolution determining β-sheet content. (B) SEM of SF mixed with two different concentrations of hpcECM to show structural changes. (C) hpcECM did not delay crystallization of SF hydrogels shown by (i) FTIR and (ii) by calculation of the increased β-sheet content after 7 days. [file 40824_2023_431_MOESM1_ESM.png]

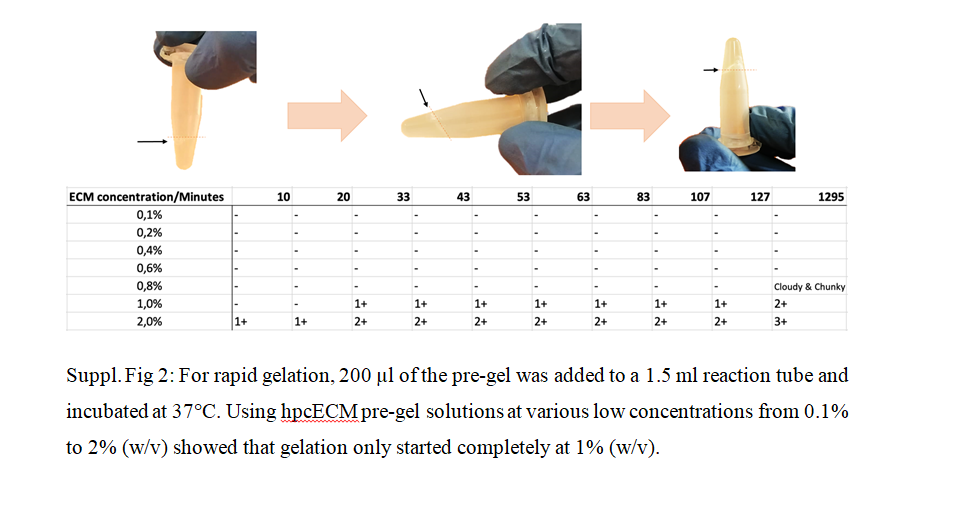

Supplement: Supplementary file 2 — Additional file 2: Figure S2. For rapid gelation, 200 µl of the pre-gel was added to a 1.5 ml reaction tube and incubated at 37°C. Using hpcECM pre-gel solutions at various low concentrations from 0.1% to 2% (w/v) showed that gelation only started completely at 1% (w/v). [file 40824_2023_431_MOESM2_ESM.png]

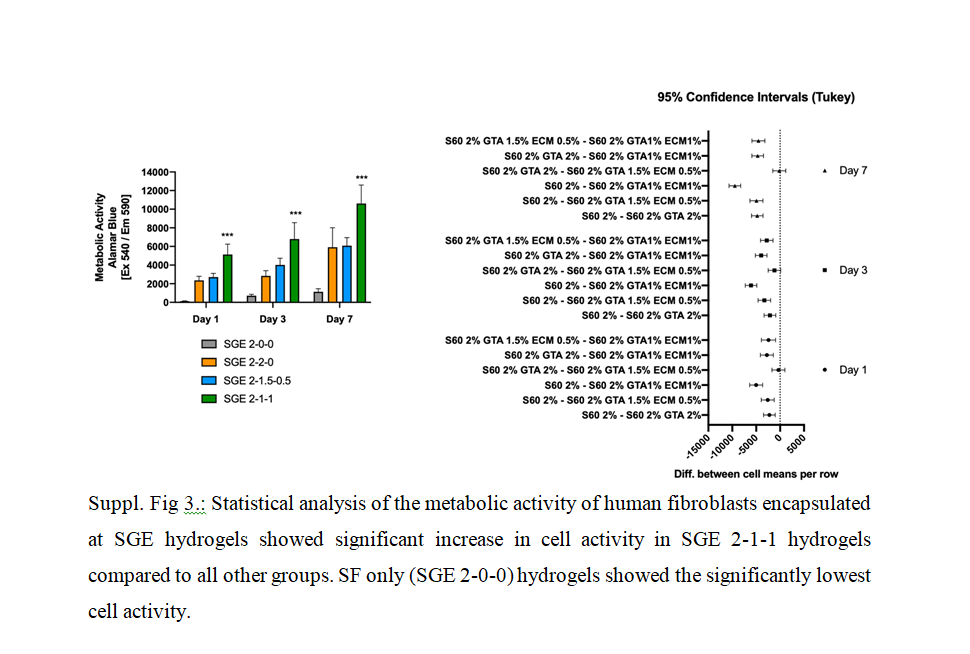

Supplement: Supplementary file 3 — Additional file 3: Figure S3. Statistical analysis of the metabolic activity of human fibroblasts encapsulated at SGE hydrogels showed significant increase in cell activity in SGE 2-1-1 hydrogels compared to all other groups. SF only (SGE 2-0-0) hydrogels showed the significantly lowest cell activity. [file 40824_2023_431_MOESM3_ESM.png]

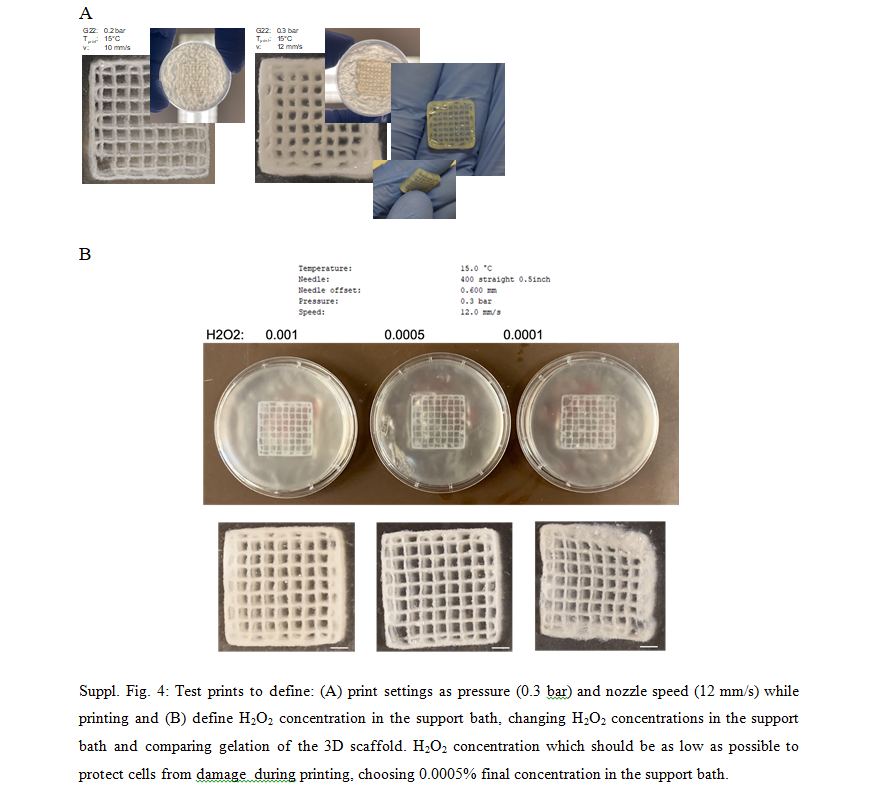

Supplement: Supplementary file 4 — Additional file 4: Figure S4. Test prints to define: (A) print settings as pressure (0.3 bar) and nozzle speed (12 mm/s) while printing and (B) define H2O2 concentration in the support bath, changing H2O2 concentrations in the support bath and comparing gelation of the 3D scaffold. H2O2 concentration which should be as low as possible to protect cells from damage during printing, choosing 0.0005% final concentration in the support bath. [file 40824_2023_431_MOESM4_ESM.png]

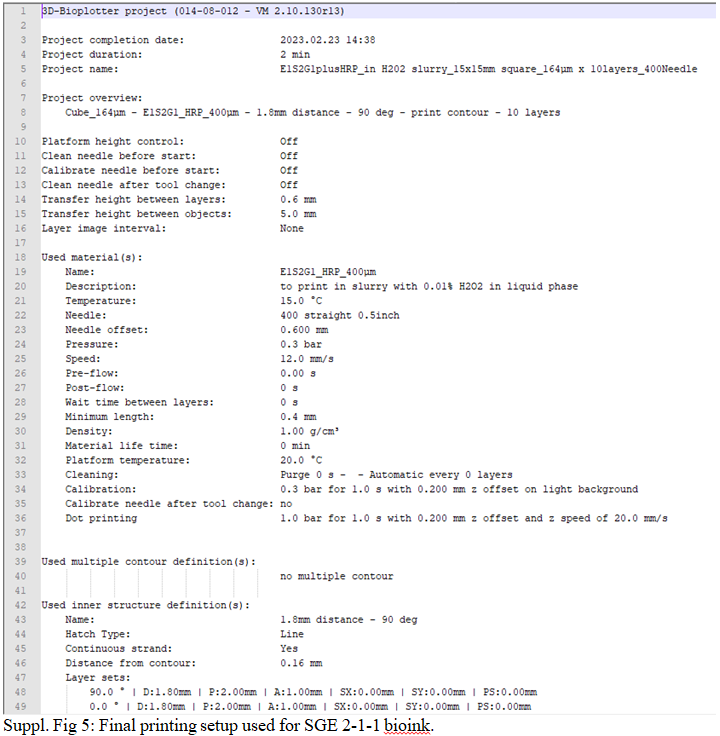

Supplement: Supplementary file 5 — Additional file 5: Figure S5. Final printing setup used for SGE 2-1-1 bioink. [file 40824_2023_431_MOESM5_ESM.png]
